# Pink Urine Syndrome: A Rare Propofol‐Associated Phenomenon in Critically Ill Patients

**DOI:** 10.1155/crcc/8811706

**Published:** 2025-09-13

**Authors:** Katrina Villegas, Ahmad Nouri, Islam Rajab, Utku Ekin, Sushilkumar Gupta, Ruth Lamm

**Affiliations:** ^1^ Department of Internal Medicine, St. Joseph’s University Medical Center, Paterson, New Jersey, USA, stjosephshealth.org; ^2^ Department of Medicine, Faculty of Medicine and Health Sciences, An-Najah National University, Nablus, State of Palestine, najah.edu; ^3^ Department of Critical Care Medicine, St. Joseph’s University Medical Center, Paterson, New Jersey, USA, stjosephshealth.org

## Abstract

Urine discoloration is a frequent clinical observation that often indicates underlying pathological or pharmacological conditions. Pink urine syndrome (PUS) is a rare phenomenon characterized by the sudden appearance of pink‐colored urine, typically attributed to the excretion and crystallization of propofol metabolites. Factors such as obesity, dehydration, and critical illness may exacerbate this condition. Although rare, PUS should be considered in critically ill patients receiving propofol sedation in the intensive care setting. This case report describes the occurrence of PUS in a 29‐year‐old male with a body mass index of 27.31 kg/m^2^, who was sedated with propofol following an overdose. The urine discoloration resolved after discontinuation of propofol and initiation of hydration therapy, emphasizing the importance of early recognition and prompt intervention. While PUS is benign, generally self‐limiting, and resolves without specific treatment, careful monitoring is essential to mitigate potential complications due to precipitation of uric acid crystals, such as urolithiasis, obstructive uropathy, and acute kidney injury, if exposure continues.

## 1. Introduction

Urine discoloration is a clinically significant phenomenon that often raises concern among patients and clinicians, as it can serve as a visible marker of underlying pathological processes or pharmacological effects [1, 2]. Pink urine syndrome (PUS), a rare and enigmatic condition, is characterized by the sudden appearance of pink‐hued urine or sediment. Unlike hematuria or pigment alterations induced by dietary factors, PUS is distinctly attributed to uric acid crystals forming pink deposits in the urine [1, 3]. While the precise biochemical mechanisms underlying this condition remain incompletely elucidated, a constellation of factors, including obesity, surgical stress, dehydration, and the administration of propofol, has been implicated in its pathogenesis [2–4]. Notably, propofol, a widely used anesthetic, is known to produce a spectrum of urine discolorations, including white, green, and, less commonly, pink, underscoring the unique biochemical interplay associated with this drug [1, 2].

PUS is exceedingly rare, with the literature largely limited to isolated case reports [3, 4]. Its incidence remains undefined, though it has been predominantly reported in specific clinical contexts, such as following propofol anesthesia or in obese postoperative patients [4, 5]. The condition’s rarity and nonspecific presentation contribute to diagnostic challenges, including the potential for misdiagnosis or unnecessary investigations aimed at ruling out more common causes of urine discoloration [1, 5]. Recognizing PUS is clinically significant, as it is a self‐limiting condition that resolves with the discontinuation of propofol and appropriate hydration. Early identification can reduce unnecessary investigations and healthcare costs while also improving patient care by preventing the overuse of diagnostic resources.

This manuscript reviews the mechanisms, risk factors, and clinical management of PUS, aiming to increase awareness and improve the recognition and management of this rare condition in clinical practice.

## 2. Case Presentation

A 29‐year‐old male with a history of polysubstance abuse presented to the emergency department (ED) after a presumed overdose. He was found unconscious on the street by emergency medical services (EMSs), who administered 2 mg of intranasal naloxone (Narcan). After regaining consciousness, the patient became agitated and required 50 mg of intramuscular ketamine for sedation.

Upon arrival at the ED, his vital signs were as follows: blood pressure 108/55 mmHg, heart rate 98 beats per minute, respiratory rate 18 breaths per minute, temperature 36.8°C, and oxygen saturation 86% on a nonrebreather mask; anthropometrics: height 175 cm and weight 83.6 kg, with a body mass index (BMI) of 27.31 kg/m^2^. Arterial blood gas analysis revealed a pH of 7.2, pCO_2_ of 55 mmHg, and pO_2_ of 88 mmHg. Laboratory studies showed a blood glucose level of 186 mg/dL, creatine kinase of 249 U/L, white blood cell count of 26,400/*μ*L, and hemoglobin of 13.1 g/dL. Urinalysis demonstrated clear and colorless urine with a pH of 5.0, specific gravity of 1.005, and no crystals. A urine toxicology screen was positive for benzodiazepines and cocaine but negative for opiates, despite the patient’s reported history of opioid use.

The patient was sedated with ketamine 150 mg and rocuronium 200 mg and intubated for airway protection, following rapid sequence intubation (RSI) protocols. He was admitted to the medical intensive care unit (MICU) for further management, with empiric intravenous antibiotics initiated to address suspected infection.

**Figure 1:**
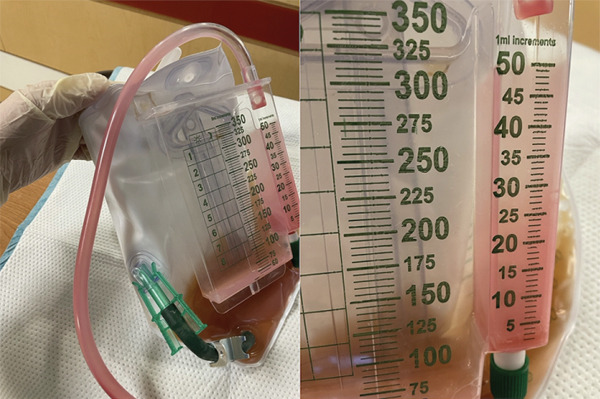
Pink‐tinged urine indicating the presence of uric acid crystals associated with propofol infusion.

During his MICU course, sedation with propofol infusion was initiated at 10 *μ*g/kg/min and titrated to 25–35 *μ*g/kg/min to maintain an initial RASS of −2 to −3. The patient developed PUS, a rare complication associated with propofol use. Bright pink discoloration was observed in his urine, urinary tubing, and collection bag (Figure 1). This condition was attributed to the excretion and crystallization of propofol metabolites. He had been on a propofol infusion for approximately 34 h, with a cumulative propofol dose of about 2.6 g. Coadministered agents included fentanyl infusion at 25–75 *μ*g/h. No midazolam, etomidate, or dexmedetomidine was used before the onset of pink urine. The propofol infusion was promptly discontinued due to the appearance of pink urine, a clinical sign suggestive of propofol‐associated PUS. Hydration was initiated with 2 L of lactated Ringer’s solution. Although the patient’s urine output had been poor over the preceding 24 h, his renal function and electrolytes remained stable. Urine microscopy was planned to monitor for crystal formation, but initial urinalysis was not feasible due to inadequate urine output. As the patient’s urine output improved with hydration, the discoloration resolved following propofol discontinuation, further supporting the diagnosis of PUS. Pink discoloration began to clear within 6–10 h after discontinuation. Close monitoring of renal function was maintained to mitigate the risk of renal injury.

Other ICU medications during the discoloration window included empiric ceftriaxone and azithromycin, stress ulcer prophylaxis with pantoprazole, and standard venous thromboembolism (VTE) prophylaxis with subcutaneous heparin. None is classically implicated in pink chromaturia.

To optimize sedation, the patient was transitioned from propofol and fentanyl to dexmedetomidine. This agent was chosen for its superior safety profile, particularly its reduced risk of delirium and respiratory depression, while still providing effective sedation. Its properties allowed the patient to remain more easily aroused and responsive to commands, which facilitated clinical assessments and communication.

On hospital Day 5, the patient was extubated and transitioned to 5 L of oxygen via nasal cannula, which was gradually tapered off. Pulmonary hygiene and physical therapy were initiated to support respiratory recovery. However, the patient left the hospital against medical advice.

Because the patient was initially found unconscious, detailed diet history immediately prior to presentation was limited. Upon regaining capacity, he denied recent ingestion of beets, blackberries, rhubarb, or food dyes, and there was no facility‐provided enteral nutrition before the onset of urine discoloration. There were no contemporaneous markers of rhabdomyolysis or hemolysis to suggest pigmenturia.

Medication history obtained from prior records and pharmacy reconciliation revealed no chronic prescriptions. On the day of presentation, the only prehospital administered agents before ICU admission were intranasal naloxone 2 mg and intramuscular ketamine 50 mg by EMS; in the ED, the patient received ketamine 150 mg and rocuronium 200 mg for RSI. No recent antibiotics (e.g., rifampin), phenazopyridine, beets/food dyes, or other pigments were identified prior to ICU admission. During the ICU course before urine discoloration was noted, continuous propofol and fentanyl infusions were used; no propofol alternatives (e.g., chlorhexidine, methylene blue, or propofol diluent substitutions) were given. These data support propofol as the most plausible contributor to the transient pink discoloration. Other medication‐related chromaturia causes (e.g., phenazopyridine orange, rifampin red/orange, metronidazole, bile pigments, amitriptyline, and indomethacin blue‐green) were not present by history or medication reconciliation.

Unfortunately, the patient experienced multiple subsequent readmissions due to recurrent drug overdoses, highlighting the ongoing challenges of substance use disorder and the need for comprehensive rehabilitation and support strategies.

## 3. Discussion

PUS is a rare and intriguing condition characterized by the sudden appearance of pink‐colored urine or sediment, without the involvement of heme, medications, or food‐based pigments [1, 3, 6]. While PUS is generally benign, its unusual presentation can cause significant clinical concern and lead to potential misdiagnosis [2]. Early recognition of PUS in critically ill patients is crucial to avoid unnecessary diagnostic workups and to reassure both clinicians and patients.

The distinctive pink hue of the urine in PUS results from the presence of uric acid crystals, which absorb pink pigments [3, 5, 6]. Although the exact biochemical mechanisms remain incompletely understood, PUS is strongly associated with increased uric acid excretion [1, 3]. It can be differentiated from hematuria by its characteristic lighter pink coloration and the presence of pink sediment that becomes more apparent after centrifugation, unlike the red coloration seen with hematuria [3, 4]. The patient presented with pink discoloration in the urine bag and tubing, leading to a diagnosis of PUS after excluding other causes.

Propofol is one of the most common factors implicated in PUS [2, 6]. Propofol is metabolized in the liver to inactive phenolic compounds, some of which are excreted in the urine [1, 2]. While propofol is also known to cause other urine discolorations, such as white urine (attributed to its oil‐in‐water emulsion) and green urine (due to phenolic metabolites), the pink discoloration seen in PUS is distinct [1, 2, 6]. Propofol has a direct effect on uric acid metabolism, significantly increasing urinary urate excretion compared to other anesthetics [2, 6, 7]. The patient was on a propofol infusion for 34 h, and the appearance of pink urine coincided with this. Additionally, propofol may indirectly induce PUS by activating the Nrf2–heme oxygenase‐1 pathway, which enhances the production of pink urinary pigments derived from bilirubin metabolism [1, 2].

Other metabolic and physiological factors, such as obesity, insulin resistance, surgical stress, dehydration, and acidic urine, further amplify the risk of developing PUS [1, 2, 5]. The patient’s BMI of 27.31 kg/m^2^ classifies him as overweight, which is a known risk factor contributing to increased uric acid production and excretion. Obesity, often associated with increased purine synthesis, leads to elevated uric acid production and excretion, contributing to uricosuria and crystal formation in the urine [2, 4]. Additionally, insulin resistance, a common feature in obesity, reduces ammonia production, leading to low urinary pH, which favors uric acid crystallization [1, 2, 6]. Low urinary pH may also result from decreased excretion of urinary glutamate [2, 4] or defects in ammoniagenesis, which are often exacerbated by insulin resistance [1, 6]. Surgical stress is another important contributor, stimulating the release of antidiuretic hormone (ADH) and corticosteroids, both of which increase uric acid excretion and promote crystal formation [2, 4, 7]. Dehydration and acidic urine in postoperative patients also elevate urinary osmolarity, further enhancing the risk of uric acid crystal precipitation [6, 7]. Medications commonly used in the perioperative period, including losartan, amlodipine, atorvastatin, and fenofibrate, may act as secondary uricosurics and exacerbate the risk of PUS [4]. Moreover, a reduction in urinary glutamate excretion, which lowers urinary pH, has been implicated in the condition [2, 4].

In critical care settings, where patients often experience metabolic stress and dehydration and are frequently administered propofol, the recognition of PUS is particularly important. Although generally a benign condition, its presentation can cause significant concern due to the overlap with other possible differential diagnoses [2, 4, 5]. Misinterpretation of PUS as hematuria, a metabolic disturbance, or an infection‐related discoloration can lead to unnecessary diagnostic tests, such as urinalysis, urine cultures, and blood work. Thus, timely recognition of PUS is essential to prevent unwarranted investigations and enable efficient management [2, 5].

There was a temporal relationship between propofol exposure and urine discoloration in this case, with resolution after cessation and hydration. While the case design cannot establish dose–response, the chronology supports an exposure‐related effect rather than cumulative toxicity.

In the absence of hematuria, infection, or heme pigments, pink urine associated with propofol most often reflects transient uricosuria with precipitation of uric acid crystals in acidic urine and resolves after hydration and stopping the inciting agent, consistent with a benign, self‐limited phenomenon [1, 4, 6]. Nonetheless, persistent exposure to the causative agent and continued low urinary pH could theoretically favor ongoing uric acid crystallization with downstream risk of uricosuric stone formation and obstructive uropathy [2, 5, 7]. In the long term, this could lead to kidney damage if left unaddressed [1].

Management strategies for PUS focus on addressing the underlying causes and mitigating risk factors. In the case of propofol‐related PUS, stopping the propofol infusion, if possible, and providing intravenous fluids, such as lactated Ringer’s solution, to promote hydration and increase urine flow are key interventions [2, 5, 7]. Potential complications if propofol is continued despite ongoing pink urine include sustained uricosuria with crystal aggregation, uric acid nephrolithiasis, and, rarely, postobstructive acute kidney injury. Early drug cessation and hydration mitigate these risks. Discontinuation of propofol and hydration with 2 L of lactated Ringer’s solution in our patient led to resolution of the pink urine, demonstrating the effectiveness of these interventions. In cases where propofol cannot be stopped, it may be necessary to switch to another sedative [5]. Alkalinization of urine by using bicarbonate may help to prevent pigment precipitation and avoid complications [1, 5]. Close monitoring of renal function, urinary output, and pH is crucial in critically ill patients, as they are at heightened risk for complications related to fluid balance and renal function [1, 4]. Although the patient had poor urine output prior to hydration, renal function remained stable with close monitoring.

PUS, though rare and generally benign, presents significant diagnostic challenges due to its striking presentation and potential for misdiagnosis. This condition is a recognized side effect of propofol use, and awareness among clinicians is crucial to avoid unnecessary diagnostic evaluations and reassure patients. Early recognition and intervention, including discontinuation of propofol, adequate hydration, and renal monitoring, are critical to preventing renal injury and ensuring optimal patient care.

This case underscores the importance of considering PUS in critically ill patients receiving propofol, particularly when faced with unexplained urine discoloration. It also highlights the multifactorial origins of PUS, including obesity, surgical stress, and metabolic imbalances, as well as the unique challenges posed by managing patients with polysubstance abuse. A multidisciplinary approach addressing both the medical and psychiatric aspects of care is essential in such cases. Further research could explore the prevalence of PUS and potential genetic or biochemical predispositions to its development.

Recognizing PUS early not only improves patient safety but also enhances cost‐effectiveness in clinical practice, particularly in resource‐limited settings where unnecessary diagnostic procedures can be a significant burden.

## Ethics Statement

An ethical review is not necessary because this is a case report.

## Consent

Written informed consent was obtained from the patient for the publication of this case report and any accompanying images. The patient was informed that identifying information would be removed to ensure anonymity and consented to the use of the patient’s medical history for educational and research purposes.

## Conflicts of Interest

The authors declare no conflicts of interest.

## Author Contributions

All authors performed the literature review and contributed to the writing, the final editing, and the collection of the patient’s clinical data.

## Funding

No funding was received for this manuscript.

## Data Availability

The data that support the findings of this study are available from the corresponding author upon reasonable request.
